# A Novel Poly-N-Epoxy Propyl Carbazole Based Memory Device

**DOI:** 10.3390/polym13101594

**Published:** 2021-05-15

**Authors:** Ahmed. N. M. Alahmadi, Khasan S. Karimov

**Affiliations:** 1 Electrical Engineering Department, Umm-Al-Qura University, Makkah 21955, Saudi Arabia; 2 Ghulam Ishaq Khan Institute of Engineering Sciences and Technology, Topi 23640, Khyber Pakhtunkhwa, Pakistan; khasan@giki.edu.pk

**Keywords:** memory device, organic semiconductors, poly-N-epoxy-propylcarbazole, tera-cyanoquino-dimethane

## Abstract

Generally, polymer-based memory devices store information in a manner distinct from that of silicon-based memory devices. Conventional silicon memory devices store charges as either zero or one for digital information, whereas most polymers store charges by the switching of electrical resistance. For the first time, this study reports that the novel conducting polymer Poly-N-Epoxy-Propyl Carbazole (PEPC) can offer effective memory storage behavior. In the current research, the electrical characterization of a single layer memory device (metal/polymer/metal) using PEPC, with or without doping of charge transfer complexes 7,7,8,8-tetra-cyanoquino-dimethane (TCNQ), was investigated. From the current–voltage characteristics, it was found that PEPC shows memory switching effects in both cases (with or without the TCNQ complex). However, in the presence of TCNQ, the PEPC performs faster memory switching at relatively lower voltage and, therefore, a higher ON and OFF ratio (I_ON_/I_OFF_ ~ 100) was observed. The outcome of this study may help to further understand the memory switching effects of conducting polymer.

## 1. Introduction

Polymer-based electronic devices have been a popular research area in recent decades due to their unmatched properties, such as light weight, flexibility, low cost, scale-ability, low-temperature processing, tenability, etc. [1,2,3,4]. As a result, these devices are an excellent choice for light-emitting diodes, solar cells, low-cost RFID, sensors, and many other electronic devices [5,6,7,8]. For a number of applications, such as mobile phones, smart watches, and other electronic devices, low-cost and flexible memory devices are essential [9,10]. Therefore, polymer-based memory devices, compared to Si-based memory devices, represent a new trend that not only exploits the reported advantages of polymer, but also improves the storage capacity of memory space for future disposable electronic devices [10].

For this purpose, many semiconducting polymer materials have been previously reported to show capacitive, resistive, and transistor-based memory effects; among these, carbazole-containing materials (e.g., poly-N-vinylcarbazole, PVC) are gaining considerable attention [11,12,13]. The observed resistive memory response of PVK thin film is accounted due to the change in the electrical resistivity as a function of the applied electric field [14,15,16]. The possible mechanisms for resistive switching memory in conductive polymer can be approximately categorized into three types: reduction/oxidation, electronic, and thermal. In the reduction/oxidation type of resistive memory device, the ions are migrated towards the corresponding electrodes due to a series of electrochemical reactions, and hence form a low resistive (ON) and high resistive (OFF) conducting path between electrodes under the influence of the applied electric field [14,17]. Similarly, for the electronic type of resistive memory device, the injected carriers are trapped (high resistance) and released (low resistance) during the charge transport process. Electronic charge trapping mechanisms are further classified as charge trapping inside the band gap, bulk charge trapping in the presence of the space charge, and charge trapping at metal–polymer interfaces. By comparison, in the thermal type of resistive memory device, the memory switching effects are originated by the formation and rupture of the conductive filamentary path initiated by the local Joule-heating effects [14,18].

In the trapped space charge limited current model, it is generally accepted that both the positional and energetic traps have the capability to capture free charge carriers, which are distributed throughout the polymer layer and degrade the free carrier mobility (high resistance, OFF state), particularly at a lower operating voltage. In a higher applied electric field, many free carriers overcome the trap barrier potential to form a space charge, increasing the mobility and hence the conductivity (ON state) of the polymer thin film [15,19,20,21,22,23]. Such resistive ON and OFF behavior can be observed as hysteresis due to the film’s current–voltage characteristics. Therefore, the trapped space charge effect plays a vital role in defining the memory effect for many organic and polymer-based electronic devices [24].

Organic molecules, and particularly TCNQ-based charge transfer complexes, have a long history of use in memory and other electronic devices [25,26,27,28], and the highly stable and reliable memory response was noted for a Cu/Cu-TCNQ/Al device, as reported by numerous researchers [29,30]. Therefore, in this study, a novel Cu/PEPC-TCNQ/Ag device was fabricated and investigated. It was observed that the device shows a high rectification ratio (I_ON_/I_OFF_ ~ 100) with extended retention time, which is highly suitable for memory devices.

## 2. Device Fabrication

**Figure 1:**
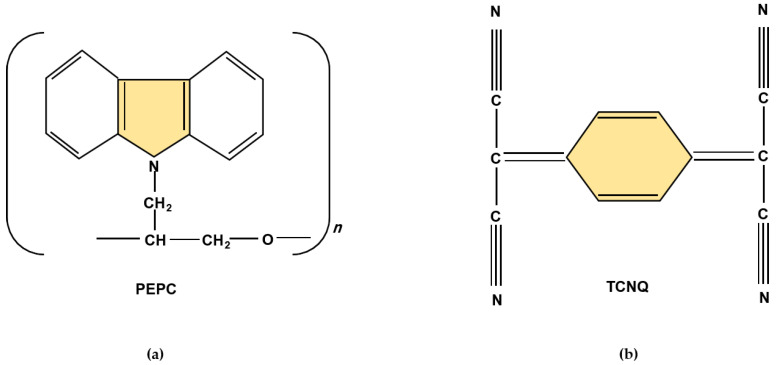
Molecular structure of (**a**) poly-N-epoxy-propylcarbazole (PEPC); and (**b**) 7,7,8,8-tetra-cyanoquino-dimethane (TCNQ).

**Figure 2:**
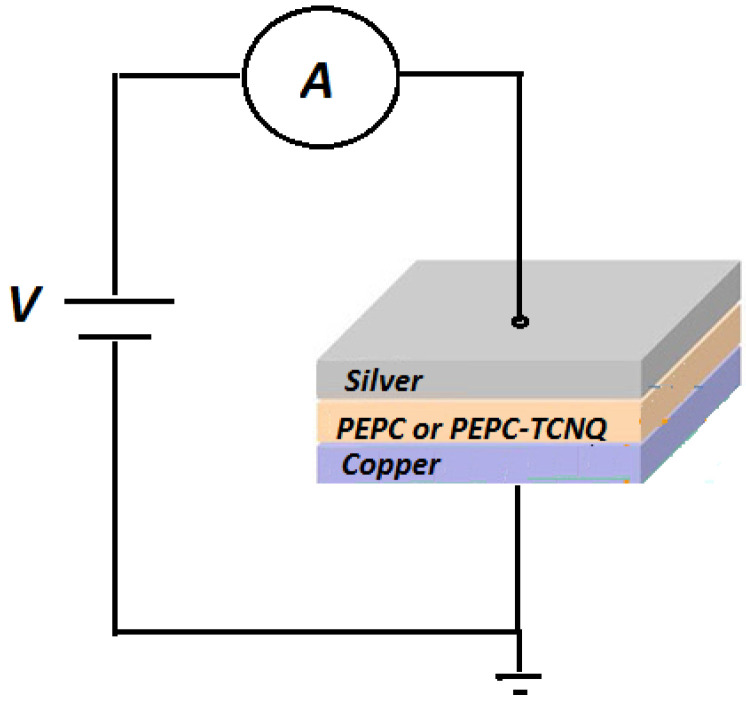
A cross-sectional view of a metal/polymer/metal diode fabricated using p-type PEPC, with or without TCNQ, as a Cu/PEPC/Ag- and Cu/PEPC-TCNQ/Ag-based memory device; for forward bias, the positive and negative terminals of the battery were connected to the top (Ag) and bottom (Cu) electrodes, respectively, for both devices.

For the resistive memory device, a simple metal–polymer–metal like diode structure was fabricated at room temperature. For the active polymer layer, PEPC and TCNQ complex materials were selected, based on the advantages discussed above. The chemicals TCNQ (CAS number: 1518-16-7; molecular weight: 204.19; empirical formula (Hill Notation): C_12_H_4_N_4_) and tetrahydrofuran (CAS number: 109-99-9; molecular weight: 72.11; empirical formula (Hill Notation): C_4_H_8_O) were purchased from Sigma–Aldrich (Karachi, Pakistan), and PEPC was locally developed, for which detailed information can be found elsewhere [12,13,28]. The purchased chemicals were used without any further purification. The molecular structure of PEPC (1400 amu) and TCNQ is shown in Figure 1a,b, respectively. Generally, the doping of PEPC with TCNQ makes a charge transfer complex, where PEPC behaves as an electron donor, whereas the low-molecular-weight organic material TCNQ behaves as an electron acceptor [28]. The conductive TCNQ was obtained after successive processes of re-crystallization with acetonitrile solvent. Because both PEPC and TCNQ are soluble in tetrahydrofuran as an organic solvent, a solution was made between PEPC and TCNQ (4:1 ratio) with 8% by weight in the solvent tetrahydrofuran. Thin films of both PEPC and PEPC-TCNQ solution were deposited by the spin-coating method (1000 rpm, 30 s) over 99.99% Cu substrate, separately. The thickness of the films was in the range of 500 nm–1.2 µm, and the average surface area of the films was in the range of 1.6–2.1 cm^2^. For another electrode, highly conductive silver paste was deposited onto the PEPC-TCNQ thin films. For characterization, three samples were fabricated for Cu/PEPC/Ag and Cu/PEPC-TCNQ/Ag devices, and the median response (which was very close to the average response for most cases) among the three samples was selected for the further analysis that led to the conclusions. A schematic cross-section of the sample is shown in Figure 2. Using the hot probe method, it was observed that both PEPC and PEPC-TCNQ behave as a p-type semiconductor [28]. For forward bias current–voltage characteristics, the positive and negative terminal of the battery was connected to the top electrode (Ag) and bottom (Cu) electrode, respectively, for both devices, as shown in Figure 2.

## 3. Results and Discussion

**Figure 3:**
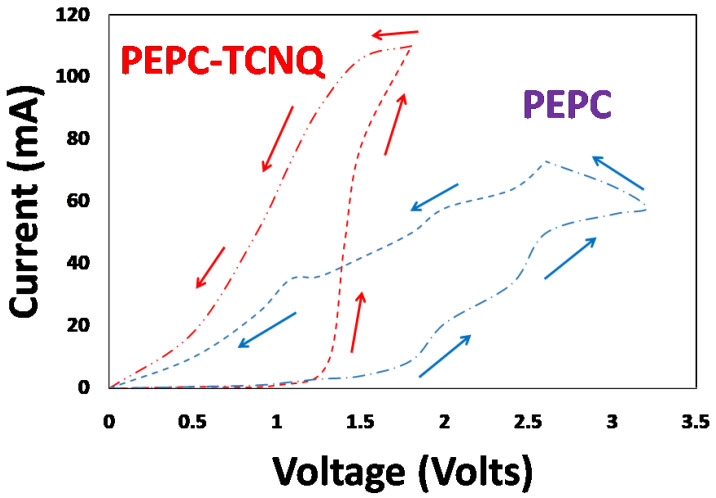
Current–voltage characteristics of Cu/PEPC/Ag and Cu/PEPC-TCNQ/Ag diodes measured at room temperature, showing a typical memory hysteresis curve for both devices. The sweeping path in the figure is shown by the arrow directions.

The current–voltage characteristics of both the Cu/PEPC/Ag and Cu/PEPC-TCNQ/Ag devices were measured at room temperature, as shown in Figure 3. Because we were mainly interested in qualitatively determining and comparing the memory effects for the PEPC and PEPC-TCNQ devices, we measured the current–voltage hysteresis loop for both devices at a sweeping rate of 100 mV/sec for simplicity. The figure shows that both devices exhibited electrical switching effects in both forward and reverse cycles. In the first cycle, the applied voltage increased in the forward bias from zero to 3.25 V for the Cu/PEPC/Ag devices, and from 0 to 1.5 V for the Cu/PEPC-TCNQ/Ag device, whereas in the second cycle, the applied voltage decreased in the forward bias from 3.25 and 1.5 V to zero voltage, respectively. The sweeping rate for both directions of the cycle was maintained at 100 mV/sec without any hold time. Both devices followed different current paths for different cycles, clearly demonstrating an electrical memory effect (transition from high resistance to low resistance) for both devices. The transition from high resistance state to low transition state was observed at ~2.5 V for the Cu/PEPC/Ag device, whereas a sharp transition was observed at 1.5 V for Cu/PEPC-TCNQ/Ag. This result indicates that doping of PEPC with TCNQ improves the electrical bi-stability of the memory behavior.

**Figure 4:**
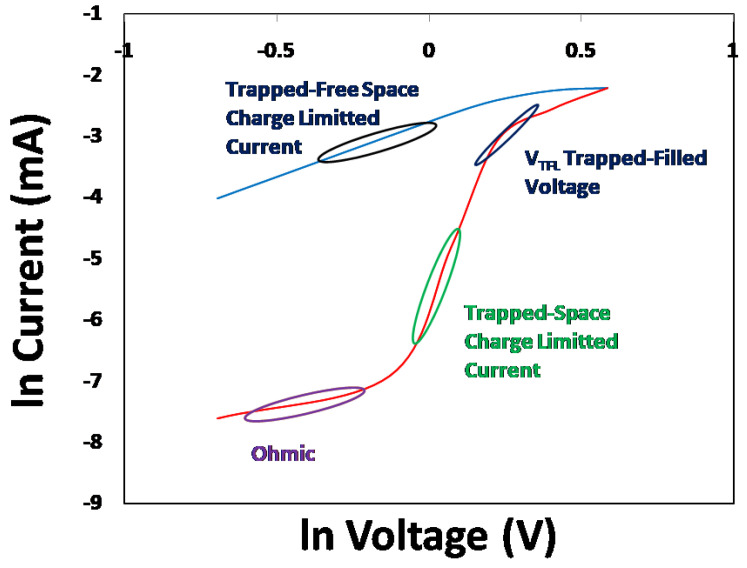
ln Current vs. ln Voltage characteristics of the Cu/PEPC-TCNQ/Ag diode, which clearly shows possible charge transport mechanisms as: (1) ohmic region; (2) trapped-space charge region; (3) trapped-filled voltage region; and (4) trapped-free space charge region.

To further investigate the likely switching mechanism of the Cu/PEPC-TCNQ/Ag device, the ln(current) vs. ln(voltage) characteristics were further explored. Four well-defined charge transport regions were found as follows: (1) ohmic region; (2) trapped space charge region; (3) trapped filled voltage region (VTFL); and (4) trap-free space charge region [31,32,33], as shown in Figure 4. At an early stage, the devices follow ohmic response, leading to the trapped space charge limited current. This can be explained by the effects of mobility on charge concentration (holes) in the devices. With increasing voltage, the charge density increased. The sharp increase in the charge mobility with the hole density due to the trap filling was directly confirmed by [34]. By further increasing the voltage, the device reached a trapped-free region after passing through the trapped-filled voltage, as shown in Figure 3. The transformation of the trapped-space charge region to the trapped-free region is mainly accountable for the switching response of the Cu/PEPC-TCNQ/Ag device.

**Figure 5:**
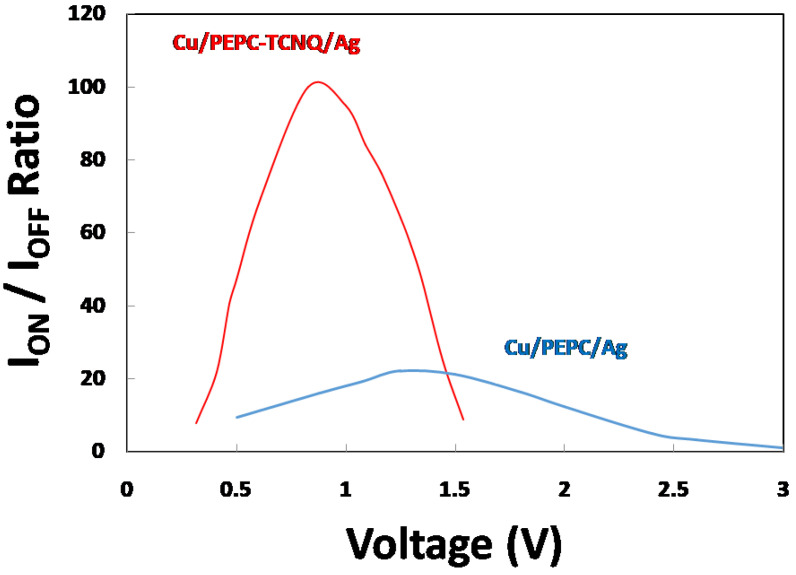
I_ON_ and I_OFF_ ratio (I_ON_/I_OFF_) for Cu/PEPC/Ag and Cu/PEPC-TCNQ/Ag devices as a function of applied voltage.

The ON–OFF current ratio (I_ON_/I_OFF_) is an important factor to define the performance of memory devices and can be estimated from the resistive hysteresis of the current–voltage response of memory devices. Because the resistive hysteresis is originated by the random trapping and de-trapping of charged carriers inside the bulk region of the polymer, the ON–OFF ratio indicates the dynamic behavior of charged carriers. Hence, a high value of the ON–OFF current ratio indicates fast switching transition, whereas a low value of the ON–OFF current ratio reveals a slow switching transition inside the bulk region of the polymer. Figure 5 shows the ON–OFF current ratio for both Cu/PEPC-TCNQ/Ag and Cu/PEPC/Ag memory devices as a function of applied voltage. The ON–OFF current ratio increases with increasing voltage up to a certain voltage with a maximum ON–OFF ratio; then, the given ratio begins to decrease as voltage increases further for both memory devices due to the complex dynamic behavior of deep-level traps. However, the domain of the voltages and the range of the ON–OFF current ratios are different for the two memory devices, as shown in the figure. The maximum and steeper I_ON_/I_OFF_ ratio (~100) are observed for the Cu/PEPC-TCNQ/Ag device at nearly 0.85 V, whereas the same maximum and broad I_ON_/I_OFF_ ratio (~23) is observed for the Cu/PEPC/Ag device at 1.4 V. The figure clearly demonstrates that PEPC-TCNQ shows very fast memory switching with an excellent I_ON_/I_OFF_ ratio compared to the PEPC-based memory device [35]. Traps for PEPC-TCNQ are simply defects or structural disorders in PEPC, which can be formed during the device fabrication process due to many known and unknown reasons, such as external or internal impurities and chemical defects [36]. It is generally accepted that the traps are energetically distributed between HOMO (highest occupied molecular orbital, valence band) and LUMO (lowest unoccupied molecular orbital, conduction band), and cause the electrical properties of polymer-based electronic devices to be degraded. Because the energy band gap of PEPC is much higher than the TCNQ energy band gap, TCNQ molecules may offer favorite sites for charge carriers hopping inside PEPC, which helps to improve the charge transport process at the applied voltage. Therefore, a visible improvement in current–voltage characteristics was observed for the Cu/PEPC-TCNQ/Ag device compared to the Cu/PEPC/Ag device.

In the trapped-space charge region, when applied voltage is gradually increased from the ohmic region, the injected hole charges from the Cu electrode are caught by traps and are no longer available as free carriers, and, overall, the device offers high resistance (OFF state) to the flow of charges. By continuously increasing voltage in this region, traps are continuously filled and a stage is reached when nearly all traps are filled by injected carriers. Because filled traps do not play any further significant role in the charge transport mechanism, this stage is called the trapped filled voltage point. After this stage, both PEPC with or without TCNQ behaves like a trapped-free space charge limited current and offers very low resistance (ON state), yielding a higher I_ON_/I_OFF_ ratio, as shown in Figure 5. However, the striking difference observed is that, for PEPC-TCNQ, the trapped-free region is achieved at a much earlier voltage (1.5 V) compared to PEPC (3.25 V), which clearly demonstrates that TCNQ improves the overall conductivity of PEPC for the ON state with fast switching for the Cu/PEPC-TCNQ/Ag memory device.

**Figure 6:**
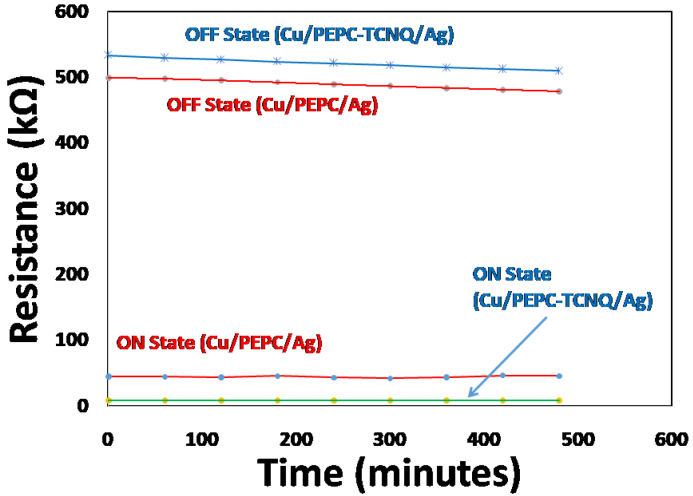
Degradation of ON state and OFF state resistance as a function of time for Cu/PEPC/Ag and Cu/PEPC-TCNQ/Ag devices after heating and holding the sample at 60 °C for ~1 h duration.

Similar to the fast switching characteristics, the stability in terms of retention of the ON and OFF states of the memory device for a longer period is another important parameter required for the performance of polymer memory devices. Therefore, the retention ON and OFF state resistance was investigated, measured from the current–voltage characteristics. For this purpose, the samples were heated up to 60 °C and held for 15 min to achieve thermal equilibrium. After thermal equilibrium, the resistance was measured for up to approximately 8 continuous hours (480 min) under the same laboratory environmental conditions. In addition, the temperature was continuously monitored to maintain it at 60 °C throughout the experiment for both devices. The output results are shown in Figure 6. Therefore, the marker points in Figure 6 correspond to the resistance of both memory devices in the ON/OFF states as a function of the same time interval. These resistance measurements were carried out in a very similar way to that discussed in Figure 3, but the resistances were calculated at 60 °C (1 volts) as a function of ageing time. The figure clearly shows that both ON state (relatively low) and OFF state (relatively high) resistance demonstrate nearly negligible degradation for both devices, illustrating the excellent stability of the Cu/PEPC-TCNQ/Ag- and Cu/PEPC/Ag-based memory devices under the given conditions.

## 4. Conclusions

To investigate memory switching effects, the electrical characterization of a single layer memory device (metal–polymer–metal) containing PEPC doped with and without TCNQ charge transfer complexes was performed for Cu/PEPC/Ag and Cu/PEPC-TCNQ/Ag memory devices. From the current–voltage characteristics, it was observed that both devices showed memory switching effects. However, the doping of PEPC with TCNQ improved the quick response of the memory device, resulting in a high ON and OFF ratio (I_ON_/I_OFF_ ~ 100). This memory switching effect may be due to the observed space charge limited behavior in the presence of trap distributions for both devices. Finally, both devices demonstrated a high degree of retention stability for the required memory operations.

## Data Availability

The data presented in this study are available on request from the author.
